# Assessment of Biophilic Design in Educational Corridors and Stairwells Using fNIRS and GSR with Generative AI Stimuli

**DOI:** 10.3390/s26030985

**Published:** 2026-02-03

**Authors:** Ji-Yeon Kim, Sung-Jun Park

**Affiliations:** Department of Architectural Engineering, Keimyung University, Daegu 42601, Republic of Korea; jyk597@kmu.kr

**Keywords:** biophilic design, educational space, corridor and stairwell, fNIRS, GSR, neurophysiological assessment, generative AI

## Abstract

In contemporary educational spaces, circulation spaces such as corridors and stairwells are central to students’ daily experience, yet their capacity to serve as therapeutic environments remains underexplored. This study quantitatively evaluated the physiological and neurocognitive impacts of Biophilic Design (BD) in these circulation spaces. Thirty university students experienced immersive virtual scenarios of corridors and stairwells that integrated four BD elements—weather & view, plants & landscape, material & texture, and forms & shapes—while prefrontal cortex (PFC) activity and stress responses were simultaneously captured using functional Near-Infrared Spectroscopy (fNIRS) and Galvanic Skin Response (GSR). Results showed that BD conditions produced significantly greater stress reduction, reflected in lower GSR, compared with non-BD conditions. fNIRS analyses further indicated enhanced PFC activation, with spatially differentiated patterns that varied by circulation space type and by specific BD elements. Collectively, these findings offer empirical neurophysiological evidence that applying BD to educational circulation spaces can mitigate stress and foster psychological stability, thereby providing a robust basis for evidence-based strategies to create healthier, cognitively supportive learning environments.

## 1. Introduction

### 1.1. Research Background

In contemporary higher education, learning environments function not only as physical infrastructures but also as critical ecological frameworks that support students’ psychological well-being and cognitive performance [1]. Circulation spaces such as corridors and stairwells, traditionally conceived as utilitarian passages, in fact serve as transitional environments where students engage in spontaneous social interactions, regulate emotions, and cultivate peer relationships [2].

While substantial research has examined static learning environments such as classrooms and libraries, empirical investigations into the psychological and physiological impacts of dynamic, everyday interactional spaces like corridors and stairwells remain remarkably limited. This research gap is particularly critical for university students, a demographic undergoing a significant developmental transition and confronting a multitude of academic and social stressors [3,4,5]. Indeed, the mental health challenges faced by university students represent a growing global concern, escalating in both prevalence and severity [6,7]. Consequently, there is a compelling need to reconceptualize transitional spaces within university campuses as therapeutic environments and to explore tangible design strategies for their realization.

In the fields of architecture and environmental psychology, biophilic design (BD) has recently gained prominence as a strategy for fostering restorative environments. The integration of natural elements has been shown to promote emotional stability and recovery while simultaneously enhancing cognitive stimulation and spatial orientation [8]. Within educational settings, BD further contributes to students’ spatial satisfaction and concentration [9]. Yet, much of the existing research has relied heavily on subjective assessments such as surveys and observations, with limited efforts to objectively validate its impact through neurophysiological evidence. Furthermore, empirical investigations into transitional spaces including corridors and stairwells are nearly absent, resulting in a critical knowledge gap regarding their therapeutic potential in educational environments.

This study aims to bridge this gap by distinguishing the effects of BD across these two distinct spatial typologies. Corridors, characterized by horizontal movement and longer sightlines, may primarily serve a restorative function, offering opportunities for mental recovery and stress reduction. In contrast, stairwells demand greater cognitive load for vertical navigation and balance, suggesting that BD interventions in these spaces might more profoundly influence cognitive engagement and attentional processes. To objectively test this, we utilize neurophysiological indicators: Galvanic Skin Response (GSR) to measure autonomic stress responses, providing a direct correlate for Stress Reduction Theory (SRT), and functional Near-Infrared Spectroscopy (fNIRS) to capture prefrontal cortex activation, offering insights into the cognitive mechanisms described by Attention Restoration Theory (ART). This methodological approach is consistent with the principles of Evidence-Based Design (EBD) and is significant for its potential to provide a scientific foundation for the design of educational facilities. Consequently, this dual-metric, spatially differentiated approach allows for a deeper theoretical synthesis, providing a robust foundation for designing healthier and cognitively supportive educational environments.

### 1.2. Objectives and Hypothesis

The purpose of this study is to empirically investigate the neurophysiological effects of BD in the corridors and stairwells of university educational facilities on learners. To this end, the following research questions were established:

**H0.**  *Biophilic design (BD) conditions will not show significantly different effects on learners’ emotional stress (GSR) or cognitive activation (fNIRS) compared to Non-BD conditions*.

**H1.**  *Biophilic design will significantly decrease learners’ emotional stress (GSR) and significantly increase cognitive activation (fNIRS) differently based on the space type (corridors, stairwells)*.

**H2.**  *Some of the four elements of biophilic design (Weather & View, Plants & Landscape, Material & Texture, Forms & Shapes) will promote the activation of specific brain regions (Dorsolateral Prefrontal Cortex [DLPF], Frontal Polar Area [FPA], Orbitofrontal Cortex [OFC], and Broca’s Area [BA]) more strongly than others*.

**H3.**  *The effects of biophilic design will differ based on the space type*.

▪ In corridors, emotional stability and restorative responses will be prominent.
▪ In stairwells, attention and cognitive arousal responses will be relatively stronger.

## 2. Theoretical Insight and Background

### 2.1. Psychological and Functional Dimensions of Circulation Spaces

Corridors and stairwells have traditionally been viewed solely as functional pathways for the movement of people within a building. However, in contemporary educational facilities, these spaces are being reevaluated as key locations that mediate students’ experiences and interactions. Physical elements such as spatial width, openness, and lighting directly impact psychological stability and cognitive processes. Thus, these thoroughfares are evolving from simple paths into multidimensional educational transition spaces [10].

Specifically, corridors and stairwells function as social infrastructure that connects major learning areas while fostering informal interactions among students [11,12]. Corridors serve as venues for forming free social relationships through brief stops, chance encounters, and informal conversations that occur during movement between classrooms [13,14], while stairs can extend beyond simple vertical movement to become community spaces for rest, communication, and informal learning when combined with seating or multipurpose halls [2]. Therefore, it is necessary to understand these spaces not as mere pathways but as educational transition spaces that influence the holistic development and experiences of students [15].

Furthermore, corridors and stairwells are deeply involved in the process of spatial cognition. By navigating these transitional spaces daily, students form a cognitive map of the school building, which in turn provides a sense of psychological security and orientation [16]. However, cognitive load varies depending on the type of space. Corridors, where horizontal movement is dominant, allow for more time to explore the surrounding environment, whereas stairs, which require vertical movement and balance, may limit the cognitive processing of environmental stimuli [17]. Considering these multidimensional characteristics, BD is evaluated as an effective strategy to maximize the educational and social value of these transitional spaces and to support students’ psychological well-being and holistic growth.

### 2.2. The Healing and Cognitive Effects of Biophilic Design

#### 2.2.1. Biophilic Design

Biophilic Design (BD) is an architectural approach integrating “biophilia”—the human instinct to connect with nature—into the built environment [18,19]. Throughout most of human evolution, people lived in natural settings, and today, a connection with nature remains essential for maintaining physical and mental health [20]. BD therefore aims to create high-quality habitats that satisfy fundamental human needs, improving health, productivity, and quality of life, rather than merely placing plants [21]. The therapeutic and cognitive effects of biophilic design can be explained by two key theories in environmental psychology. Stress Reduction Theory (SRT) posits that exposure to non-threatening natural environments induces an unconscious positive emotional state, stabilizing physiological indicators like heart rate and blood pressure [22]. Conversely, Attention Restoration Theory (ART) explains that the “soft fascination” provided by nature helps restore mental fatigue and enhances higher-order cognitive functions [23].

Kellert [20] classified the ways humans experience nature into three categories: Direct Experience of Nature, Indirect Experience of Nature, and Experience of Space and Place. Based on this, he proposed 25 detailed attributes (see Table 1). While Kellert’s [20] framework is conceptually comprehensive, its abstract nature limits practical design application [24].

This study thus simplifies the framework by focusing on physical and visual elements relevant to architectural practice, reconfiguring it into four key components:

▪ **Weather & View** integrates light, air, weather, and external views. It involves perceiving these elements via features like windows or atriums, which provides a connection to the outside world and improves psychological stability, concentration, and creativity [20,25].
▪ **Plants & Landscape** directly introduces the “vitality” of ecological elements (e.g., plants, water) into a space [26]. Green walls, indoor gardens, and rooftop gardens contribute to restoration, biodiversity, and a creative learning atmosphere [20,26].
▪ **Material & Texture** forges a connection with nature through the visual and tactile qualities of materials like wood, stone, and leather [26]. Minimally processed materials can enhance the sense of place and create a warm, inviting atmosphere, contributing to psychological well-being [20,25].
▪ **Forms & Shapes** incorporates biomorphic and natural geometries. Applied to a building’s facade, structure, or furniture, these forms can imbue a space with vitality and enhance cognitive comfort and focus [20,25].

Accordingly, this study empirically verifies the effects of these four BD elements in educational transitional spaces using neurophysiological indicators.

#### 2.2.2. The Role of Biophilic Design in Educational Environments

Educational spaces are the primary physical environments where students spend most of their day, so the application of BD is directly linked to learner health and performance [27]. Conversely, learning environments disconnected from nature can lead to long-term health issues and reduce academic efficiency [28].

Numerous previous studies have demonstrated the educational benefits of BD (see Table 2). Natural light and views contribute to reduced stress and improved academic achievement [29,30,31], while exposure to greenery and natural elements positively impacts emotional restoration and attention enhancement [32,33,34,35]. Furthermore, natural materials and creative design strengthen a sense of place attachment and promote social and physical well-being [36]. This contributes not only to improved academic performance but also to reduced absenteeism and increased teacher retention [37].

Case studies of educational facilities also support these effects (see Table 3). Case A employed wood finishes and skylights to introduce abundant daylight and create a warm transit experience. Case B utilized a timber structure and transparent façade to maximize daylight while adopting a biomimetic stairwell design that enhanced its symbolic value. Case C provided continuous external connectivity by incorporating open corridors and stairwells. Case D combined a curved glass façade with wood finishes to achieve both natural lighting and a sense of psychological stability. Collectively, these cases illustrate how strategic use of natural light, views, and materials can transform corridors and stairwells from mere passageways into educational transition spaces that foster psychological well-being and vitality.

However, existing research on BD has concentrated on static, long-duration spaces such as hospital rooms, offices, and classrooms [38], with very few empirical studies conducted on dynamic circulation spaces like corridors and stairwells. This is likely due to the perception that the design effects in passageway-type spaces would be limited, given the short duration of use and the primary purpose of “transit.” Nevertheless, recent studies show that biophilic elements can be unconsciously perceived even during transit, triggering psychological and physiological responses [39,40].

Specifically, VR-based studies [38,39,40,41,42] studies measuring BD effects report stress-restorative effects after only 1–5 min of brief exposure. This collective evidence supports the methodological validity of studying corridors and stairwells.

### 2.3. Physiological Metrics for Environmental Stimuli

#### 2.3.1. Physiological Indicators

Human environmental experiences result from a complex interplay of cognitive, emotional, and physical responses. Therefore, to objectively determine the effects of spatial stimuli, physiological indicators that can capture these multidimensional responses are necessary. In this study, we aim to comprehensively analyze the effects of BD in transitional spaces using fNIRS to measure changes in brain activity and GSR to reflect autonomic nervous system responses.

fNIRS is a technology that quantitatively measures changes in neural activity by tracking the concentration of oxygenated hemoglobin (HbO) and deoxygenated hemoglobin (HbR) in the cerebral cortex using near-infrared light [43]. Owing to its advantages of being safe, portable, and capable of measurements in naturalistic, real-world-like settings [44], fNIRS is regarded as an effective tool for exploring the effects of the built environment on human [43,45].

However, interpreting environmental experiences solely through the brain’s cognitive activity has limitations. A holistic analysis must also consider emotional and physical responses mediated by the Autonomic Nervous System (ANS), which can be objectively measured by GSR. GSR records electrical activity on the skin’s surface, reflecting emotional arousal and stress [46]. In recent architectural research, GSR is increasingly combined with other data to evaluate user experience from multiple perspectives [47,48].

Therefore, this study aims to clarify the impact of BD on the cognitive and emotional processes of university students in transitional spaces like corridors and stairwells. This will be accomplished by integrating the analysis of cognitive activation indicators from fNIRS with emotional response indicators from GSR, providing a holistic perspective.

#### 2.3.2. The Role of the Prefrontal Cortex in Experiencing Architectural Spaces

**Figure 1:**
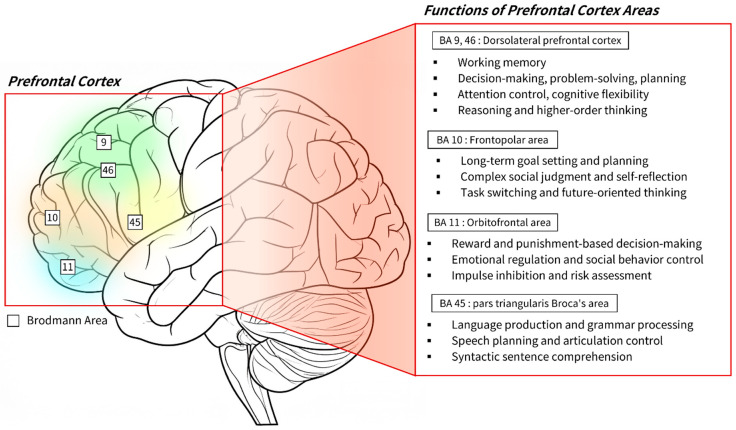
Schematic representation of prefrontal cortex subdivisions defined by Brodmann areas, accompanied by functional characterizations of each region.

This study will use fNIRS to measure the regional activity of the Prefrontal Cortex (PFC) and interpret the neurological responses to BD. The PFC is a core region for higher-order cognitive and emotional functions. Exploring its sub-regions’ activation patterns provides clues for understanding architectural experiences, such as the emotional stability and cognitive restoration BD may offer. The areas primarily analyzed in this study are as follows (see Figure 1).

First, the Dorsolateral Prefrontal Cortex (DLPFC) is responsible for executive functions such as working memory, attention shifting, and cognitive control [49], and serves as a key region for forming memory codes of visuospatial information [50]. It is also involved in emotional regulation and stress recovery, with its activation patterns varying according to task difficulty [51,52,53]. In architecture, DLPFC activation reflects how users interpret visual cues, comprehend spatial structures, and adapt to the environment. Appropriate activation suggests positive immersion, while excessive activation may indicate cognitive overload, and low activation a monotonous experience [54].

Second, the Frontal Polar Area (FPA) handles highest-order cognitive functions, such as long-term planning and exploring new possibilities [53,55]. In architecture, FPA activation reflects how users explore a space’s potential. While open and adaptive spaces stimulate creative interpretation and curiosity, monotonous and predictable environments lead to low activation, diminishing the motivation for exploration [55,56]. Thus, the FPA serves as a neurological indicator of the creative potential and opportunities for user engagement that a space provides.

Third, the Orbitofrontal Cortex (OFC) integrates sensory information to evaluate stimuli reward value, assessing spatial pleasantness and satisfaction [57,58]. In the context of architectural experience, OFC activity reflects the perceived pleasantness and satisfaction of an environment. Biophilic elements such as natural light, views, vegetation, and natural materials enhance emotional stability and spatial preference through the activation of the OFC, which underpins the user’s positive emotional experiences [59]. Conversely, excessive stimulation may elicit negative evaluations. While increased activation is linked to pleasure and aesthetic enjoyment [60,61], a lack of activation can, in turn, reflect a stable and harmonious experience [62].

Fourth, Broca’s area (BA), though traditionally linked to language [63,64] is also implicated in cognitive control and mental effort [65,66]. In the context of architectural experience, the activation of BA increases when a space’s design is not intuitively interpreted, reflecting the user’s process of resolving cognitive conflict and assigning meaning [67]. Conversely, decreased activation signifies that the spatial interpretation is intuitive and seamless, which is associated with predictable and stable environments. Conclusively, the frontal lobe mediates complex neurocognitive processes such as exploration, evaluation, and interpretation of architectural spaces. Its activation patterns therefore constitute a critical neurological indicator of how BD influences learners’ cognitive engagement and emotional stability.

## 3. Materials and Methods

### 3.1. Study Overview and Participants

This study employed a within-subject, randomized cross-over design in which all participants experienced both corridor and stairwell conditions, thereby controlling for individual differences and enabling condition-specific comparisons. Experiments were conducted in a laboratory setting under constant lighting, noise, and temperature/humidity to minimize external influences. The protocol complied with the Declaration of Helsinki and was approved by the Keimyung University Institutional Review Board (IRB No. 40525-202510-HR-059-03). Written informed consent was obtained from all participants.

Recruitment occurred over a two-month period in 2025 through posters, announcements, and emails, with inclusion criteria restricted to: (1) undergraduate or graduate students and (2) adults aged 20 years or older without major health issues. A total of 30 students participated (mean age = 23 ± 2.03 years; range = 20–30), comprising 18 males (60%) and 12 females (40%). All participants were domestic (Korean) students enrolled at the university; no international students were included in this sample.

Although the homogeneous sample limits external generalizability, it strengthened internal validity by reducing variability. To control confounding factors, participants were instructed to abstain from excessive exercise, caffeine, alcohol, and smoking prior to testing. All sessions were conducted at the same laboratory between 10:00 and 15:00 to maintain consistency.

### 3.2. Gen AI-Based Visual Stimuli Design

The visual stimuli were created using Adobe Firefly Image 4 [68] to generate SEED images of corridors (16:9) and stairwells (9:16) (See Table 4). Additionally, the parameter values and descriptions used for image generation are presented in Table 5. Adobe Firefly is recognized as a tool that can produce photorealistic renderings and broad conceptual visualizations from text prompts [69]. It is highly reliable as a research tool because it uses copyright-cleared data from sources like Adobe Stock for its training, ensuring legal and ethical stability [70,71].

The utility of Firefly has also been demonstrated in actual architecture and urban design research. Liu, et al. [72] confirmed that Firefly’s high generation efficiency and convenience were ideal for rapid prototyping during the early stages of an industrial architectural heritage renovation project. Similarly, Ozbolt and Cebeci [73] proved its potential as a tool to promote designers’ creativity by using it to explore and refine innovative forms and structural ideas for a futuristic public space design.

Table 6 was derived by comparing and integrating the BD framework [20] and visual experience classification [74] to extract key keywords for prompt generation. The table presents keywords for four elements: Weather & View, Plants & Landscape, Material & Texture, and Forms & Shapes. These keywords form the basis for the subsequent prompt structure design and visual simulation experiments. AI-based visualization for BD should prioritize reflecting visual characteristics that elicit positive emotional responses in users, rather than simply acting as an automation tool [74]. Accordingly, Weather & View includes light and view elements like diffused natural light and skylight atrium, while Plants & Landscape incorporates water features such as an indoor aquarium wall. Material & Texture focuses on tactile textures like unfinished wood and reclaimed stone, and Forms & Shapes reflects natural geometric patterns such as biomorphic ceiling forms and a fractal skylight pattern.

Table 7 demonstrates how the keywords from Table 6 were applied to the specific spatial contexts of a corridor and a stairwell, following the biophilic prompt structuring method of [74]. Each element was subdivided into four categories—Subject, Attribute, Mood, and Time & Background—enabling a shift from a purely keyword-based approach toward a more contextual and narrative-driven prompt structure. This framework represents a modified adaptation of an existing model, customized to the spatial characteristics of educational circulation spaces examined in this study. Furthermore, Table 8 presents the results of applying the proposed prompts to an actual generative AI visualization simulation. The prompts and the generated images correspond for each space, providing an intuitive view of the entire process from keyword derivation to prompt structuring and simulation.

To ensure the architectural and environmental validity of the visual stimuli, a systematic multi-stage expert evaluation was conducted. The panel comprised five experts, including two university professors specializing in architectural and spatial design, two practitioners with more than 15 years of experience in interior architecture, and one researcher with expertise in architectural visualization and environmental psychology. All five experts were of Korean nationality and currently practicing or teaching in the Republic of Korea. Each stimulus was independently assessed using a 5-point Likert scale, allowing for a rigorous appraisal of design authenticity and contextual appropriateness.

The evaluation criteria were as follows: First, the evaluation items were derived from existing research on BD [20] and architectural visualization evaluation metrics [74,75]: (1) Architectural Authenticity, (2) Contextual Appropriateness, (3) BD Element Representation, (4) Depth and perspective and (5) Visual Realism. Second, the reliability of the evaluation scale was examined using Cronbach’s alpha to assess internal consistency. In general, a Cronbach’s alpha value above 0.6 is considered acceptable [76]. In this study, the evaluation scale demonstrated adequate reliability for both datasets, with α = 0.790 for the corridor condition and α = 0.723 for the stairwell condition, confirming the consistency and robustness of the expert assessment tool. Third, to secure construct validity, a strict set of selection criteria was applied. Given the use of generative AI, stimuli were required to achieve an average score of 3.5 or higher on the ‘Visual Realism’ criterion. For the other four criteria, a higher threshold of 4.0 or higher was required. Table 9 presents the detailed evaluation results (Mean and SD) for all stimuli used in the main experiment. This verification process ensured that all selected images faithfully reflected the principles of BD and possessed high architectural and environmental validity for each space type. However, it should be noted that this validation focused on design authenticity and the representation of BD attributes, not on the cross-cultural validity of aesthetic preferences.

### 3.3. Apparatus and Experimental Procedure

All experiments were conducted in a controlled laboratory environment to minimize the influence of external factors. To simultaneously measure participants’ neurological responses (brain activity) and autonomic nervous system responses (skin conductivity), fNIRS equipment (NIRSport2, NIRx, Berlin, Germany) and a Shimmer3 GSR+ Unit (Shimmer Research Ltd., Dublin, Ireland) were used in parallel.

**Figure 2:**
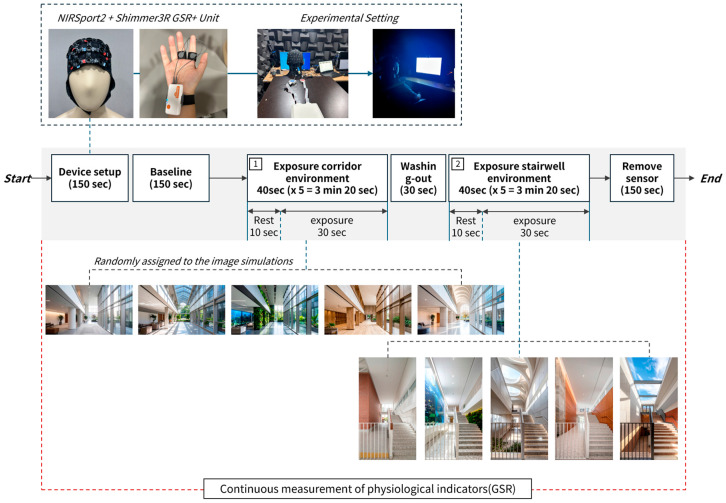
Experimental Setup. The experiment was conducted using fNIRS and Shimmer3 devices in a controlled laboratory environment.

The experimental protocol, as shown in Figure 2, proceeded through the following stages: pre-experiment setup → baseline measurement → task performance → stimulus exposure → post-experiment measurement. Throughout the entire process, external factors like noise and lighting were minimized, and constant conditions for air quality, temperature, and humidity were maintained. Before the experiment, participants were briefed on BD, and the sensors (Shimmer/fNIRS) were attached after consent was given.

First, after the sensors were attached (150 s), participants underwent a baseline measurement (150 s) in a relaxed state. They then proceeded to the task performance stage, where they were exposed to visual stimuli of different space types (corridor or stairwell). Each space type was structured in a block design, presenting five images for 40 s each (30 s of image exposure, 10 s of rest). A washing-out period of 30 s was included between image transitions to minimize interference with the measurement signals. The fNIRS device recorded real-time blood flow changes in the prefrontal cortex, while the Shimmer3 simultaneously measured GSR to record stress and arousal levels.

### 3.4. Data Processing and Statistical Analysis

The collected data were processed and analyzed using the following procedures. The fNIRS data were processed and analyzed using the MATLAB-based NirsLAB 2014.05 software. First, unrelated artifacts were removed, and a band-pass filter with a range of 0.01–0.2 Hz was applied to minimize noise and interference signals. The analysis focused on the oxygenated hemoglobin (HbO) signal. HbO is known to be more sensitive to changes in cerebral blood flow than deoxygenated hemoglobin (HbR) and is therefore a more reliable indicator of neural activity [77].

**Figure 3:**
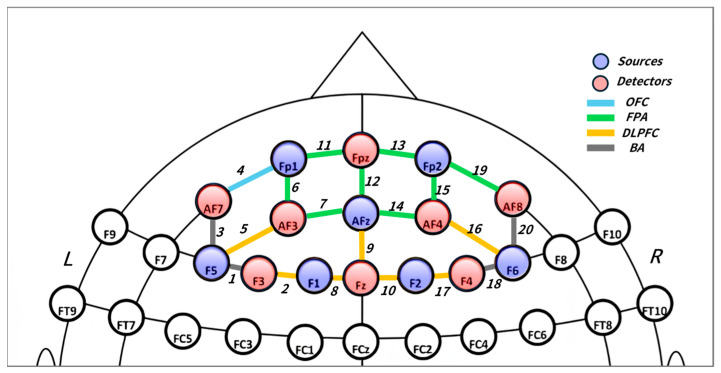
Spatial distribution of fNIRS channels over the prefrontal cortex. The optode array was positioned over the prefrontal region in accordance with the international 10–20 EEG system. Channels are categorized based on anatomical regions: dorsolateral prefrontal cortex (DLPFC), frontal polar area (FPA), orbitofrontal cortex (OFC), and Broca’s area (BA).

The measured brain area was limited to the PFC, which includes the DLPFC, the FPA, the OFC, and the BA. To precisely map each fNIRS channel to these anatomical brain regions, this study utilized the fNIRS Optodes’ Location Decider (fOLD) toolbox [78]. Figure 3 illustrates the spatial distribution of the fNIRS channels arranged according to the International 10–20 System and the PFC sub-regions to which each channel is mapped. For the GSR data, the average GSR value for each condition was calculated to analyze changes in the level of emotional arousal.

All statistical analyses were performed using IBM SPSS Statistics version 29.0.2.0. To control for individual differences, the change from baseline values (∆fNIRS, ∆GSR) was set as the dependent variable for the analysis. For each spatial type (corridor and stairwell), four paired-samples *t*-tests were conducted, comparing the Non-BD condition with each of the four BD elements (Weather & View, Plants & Landscape, Material & Texture, and Forms & Shapes). In addition, to assess differences among the four BD attributes themselves, six further paired-samples *t*-tests were performed, covering all possible pairwise comparisons. This analytical procedure was identically applied to the fNIRS data for each prefrontal cortex sub-region (DLPFC, FPA, OFC, BA) and to the GSR data. All analyses were considered statistically significant at a minimum alpha level (α) of 0.05.

Accordingly, this study recognized the issue of multiple comparisons arising from the numerous paired *t*-tests conducted. However, given the exploratory nature of investigating novel neurophysiological response patterns, an overly conservative correction (e.g., Bonferroni) was not applied. Instead, the analysis adopted a balanced approach between controlling Type I errors and enabling exploratory discovery. As noted in previous methodological discussions, conservative adjustments such as Bonferroni reduce the likelihood of false positives but simultaneously increase the risk of Type II errors, thereby diminishing statistical power [79,80]. To complement *p*-value interpretation and enhance transparency, all significant results were reported with corresponding effect sizes (Cohen’s *d*) and 95% confidence intervals (CIs) to provide a more comprehensive understanding of the magnitude and precision of observed effects.

## 4. Results

### 4.1. fNIRS Results

**Figure 4:**
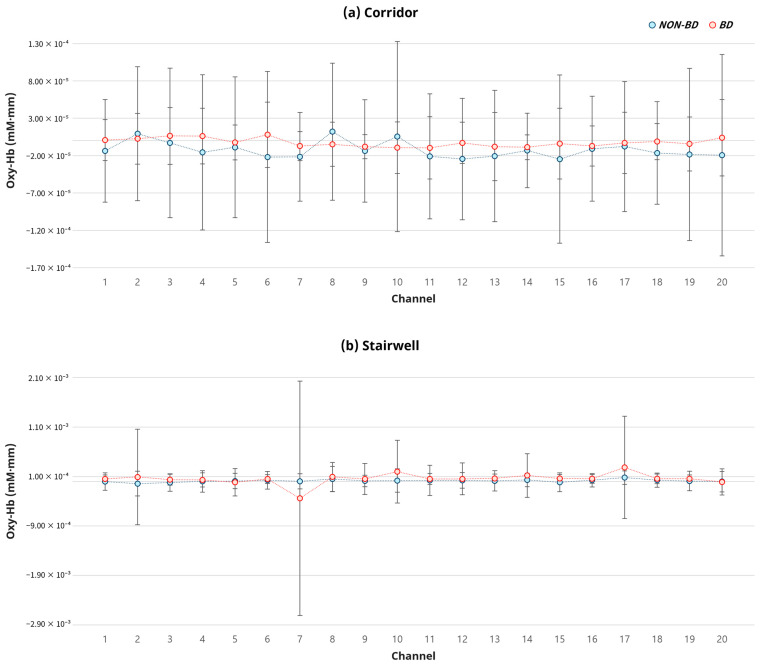
Channel-wise fNIRS results comparing biophilic and non-biophilic conditions in corridor and stairwell environments.

To analyze neural activation based on the application of BD, the average change in oxygenated hemoglobin (Oxy-Hb) across a total of 20 channels was compared (See Figure 4). In the corridor condition, Oxy-Hb decreased in both the BD and Non-BD conditions compared to the baseline. However, the magnitude of the decrease was smaller in the BD condition, maintaining a relatively higher level of activation (Figure 4a). In the stairwell condition, Oxy-Hb increased in both conditions compared to the baseline. While the BD condition showed a higher average value than the Non-BD condition, the difference was not significant (Figure 4b).

**Figure 5:**
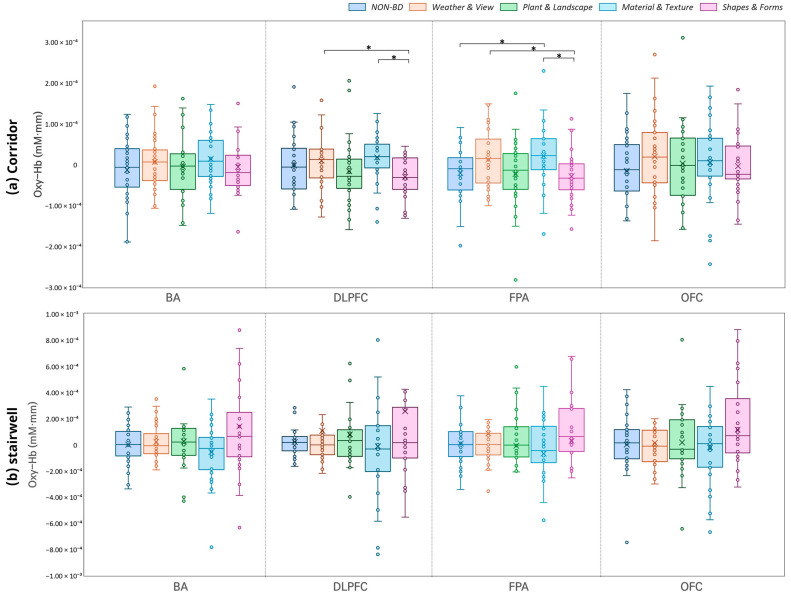
Comparison of oxy-Hb changes across brain regions (BA, DLPFC, FPA, OFC) between corridor and stair conditions by biophilic design elements (* *p* < 0.05).

The results of the analysis for each prefrontal cortex sub-region are presented in Figure 5. In the corridor condition, the BD conditions generally showed higher mean activation than the Non-BD condition, with significant differences observed between some elements. Specifically, in the DLPFC, ‘Weather & View’ showed significantly higher activation than ‘Forms & Shapes’ (M diff = 3.95 × 10^−5^, 95% CI [5.12 × 10^−6^, 7.39 × 10^−5^], t = 2.35, *p* = 0.026, d = 0.43). Similarly, ‘Material & Texture’ was also significantly higher than ‘Forms & Shapes’ (Mdiff = 4.76 × 10^−5^, 95% CI [1.22 × 10^−5^, 8.29 × 10^−5^], t = 2.75, *p* = 0.010, d = 0.50). In the FPA, ‘Material & Texture’ was higher than the Non-BD condition (M diff = −4.23 × 10^−5^, 95% CI [−7.81 × 10^−5^, −6.35 × 10^−6^], t = −2.41, *p* = 0.023, d = 0.44). Furthermore, both ‘Weather & View’ and ‘Material & Texture’ exhibited higher activation compared to ‘Forms & Shapes’ (‘Weather & View’: M diff $=4.08\times 10^{-5}$, 95% CI [4.93 × 10^−6^, 7.66 × 10^−5^], t=2.326, p=0.027, d=0.42; ‘Material & Texture’: $M\;\mathrm{diff}\;=5.02\times 10^{-5}$, 95% CI [4.52 × 10^−6^, 9.59 × 10^−5^], t=2.248, p=0.032, d=0.41). However, no significant differences between conditions were observed in the OFC and BA. In the stairwell condition, the BD conditions showed a higher mean activation than the Non-BD condition on average, but these differences were not statistically significant in any of the sub-regions.

### 4.2. GSR Results

**Figure 6:**
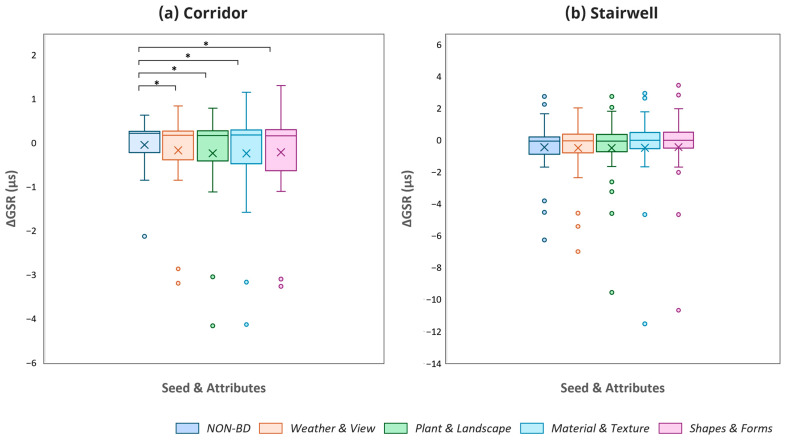
Comparison of GSR across the non-biophilic and biophilic design conditions applied within the corridor environment (* *p* < 0.05).

GSR data were analyzed in the same manner as the fNIRS data, and the results are presented in Figure 6. In the corridor condition, no significant differences were found among the BD elements themselves. However, all four BD elements showed significantly lower GSR values compared to the Non-BD condition. Specifically, statistically significant differences were confirmed for Specifically, statistically significant differences were confirmed for ‘Weather & View’ (M diff = 0.142 µS, 95% CI [0.010, 0.273], t = 2.209, *p* = 0.035, d = 0.40), ‘Plants & Landscape’ (M diff = 0.215 µS, 95% CI [0.014, 0.417], t = 2.186, *p* = 0.037, d = 0.40), ‘Material & Texture’ (M diff = 0.221 µS, 95% CI [0.001, 0.441], t = 2.053, *p* = 0.049, d = 0.40), and ‘Forms & Shapes’ (M diff = 0.190 µS, 95% CI [0.013, 0.368], t = 2.190, *p* = 0.037, d = 0.40). In the stairwell condition, the mean GSR difference between the BD and Non-BD conditions was less than 0.05 µS, and these differences were not statistically significant for any of the four elements (*p* > 0.70).

### 4.3. Summary

This study explored the effects of BD elements in corridors and stairwells on neurophysiological (fNIRS) and psychophysiological (GSR) responses. Overall, BD demonstrated positive effects on brain activation and autonomic stabilization, though the patterns varied depending on the spatial type.

First, the fNIRS results indicated that the corridor environment showed generally higher activation under BD compared to Non-BD, with significant differences observed particularly in the DLPFC and FPA. As presented in Table 8, the corridor images incorporated skylights and large glazing providing daylight and views (Weather & View), indoor planting with greenery and water features (Plants & Landscape), and textured natural materials such as wood and stone (Material & Texture). These elements appeared to act as stimuli that facilitated working memory and attentional shifting (DLPFC) as well as future-oriented exploration (FPA). In contrast, no significant differences were observed in the OFC and BA, suggesting that the corridor emphasized cognitive processing and exploratory functions rather than affective evaluation or meaning attribution. The stairwell environment showed a similar tendency, but no clear significance was found, likely due to the spatial demands associated with vertical movement.

Second, the GSR results showed that, in the corridor, all BD elements elicited significantly lower arousal levels compared to the Seed condition. This suggests that even in corridors, characterized by repetitive circulation and short stays, daylight, planting, and material textures contributed to autonomic stabilization. Conversely, in the stairwell, BD conditions tended to show lower mean values but with high variability and individual differences, which rendered the effects non-significant.

Third, hypothesis testing revealed the following: H0 was rejected in the corridor condition, as BD consistently generated significantly lower GSR and higher fNIRS activation compared to the Non-BD condition. In contrast, H0 was not fully rejected in the stairwell, where BD showed favorable trends but did not produce statistically significant differences in GSR. H1 and H3 were clearly supported in the corridor condition but only partially supported in the stairwell, while H2 was supported in terms of element-specific differential effects. In summary, the corridor demonstrated that the integration of BD elements simultaneously promoted cognitive engagement and emotional stabilization, whereas in the stairwell, the effects of BD elements were more limited.

In other words, when design elements such as daylight and views, greenery and water features, natural materials and textures, and curvilinear forms were integrated into corridors, significant improvements were observed in both cognitive engagement and emotional stabilization. By contrast, in stairwells, the same elements produced only limited effects, suggesting that BD elements were either perceived less strongly or manifested less consistently due to the functional characteristics of the space. These findings provide empirical evidence for extending SRT and ART to different spatial types, and highlight the effectiveness of incorporating daylight, views, textured natural materials, and greenery with water features as strategic design interventions particularly in corridor environments.

## 5. Discussion

This study empirically examined the differential effects of BD on learners’ cognitive and emotional responses within educational circulation spaces. The most consistent finding was a significant reduction in GSR under BD conditions in corridors, supporting SRT [22]. Brief exposure to natural cues such as light, vegetation, and material surfaces appeared to lower sympathetic activation, indicating that restorative responses can occur even during transitional movement, not only in static environments.

Beyond physiological relaxation, the results revealed deeper cognitive implications. The fNIRS analysis showed significant activation in the DLPFC and FPA under BD corridor conditions. These regions are associated with interpreting visual cues, spatial adaptation, and environmental exploration, aligning with the ART [23] and its concept of soft fascination. Natural stimuli thus promote cognitive restoration by engaging attention gently without demanding effortful focus. Accordingly, biophilic corridors may function as cognitive restorative spaces that support both physiological calmness and mental reorientation between learning tasks. In contrast, no significant differences were found in the OFC and BA, suggesting that BD stimuli activated cognition in a stable and harmonious manner rather than eliciting emotional arousal or cognitive conflict [57,58,65,66]. Participants appeared to perceive BD corridors as balanced and comfortable environments that supported a calm yet attentive state.

In the stairwell condition, these effects were less pronounced. Unlike horizontally open corridors, stairwells require vertical movement and balance, imposing higher cognitive load and limiting the perception of restorative cues. Thus, movement itself likely consumed attentional resources, reducing the ability to process natural stimuli. This finding aligns with prior research showing that restorative effects weaken under spatial confinement or forced movement [40,81].

Among BD elements, ‘Weather & View’ and ‘Material & Texture’ produced the most prominent effects. Weather & View linked indoor spaces with external natural rhythms such as daylight variation, airflow, and cloud movement, enhancing visual openness and psychological stability [20,25]. Such stimuli simultaneously reduced sympathetic arousal, as reflected by lower GSR, and promoted attentional recovery. Material & Texture provided indirect contact with nature through familiar tactile and visual qualities of materials such as wood and stone [26]. The subtle irregularities and fractal-like patterns of natural surfaces created organized complexity, which supported attentional restoration and induced emotional warmth [29,33].

Together, these elements maintained sensory stability while providing appropriate cognitive stimulation, contributing to both attentional recovery and physiological balance. These findings redefine the architectural role of corridors. Rather than mere passageways, corridors act as cognitive transition zones, mediating shifts between learning, social interaction, and rest. Physiologically, they relieve tension; cognitively, they facilitate a mental reset that prepares users for subsequent activities. Therefore, incorporating natural light, external views, and natural materials into corridor design should be considered an evidence-based strategy for enhancing cognitive and emotional well-being.

Based on these results, the concept of transitional restoration can be extended beyond educational facilities to other high-stress environments such as hospitals, offices, and mixed-use complexes. In spaces characterized by frequent movement and sensory transitions, strategic integration of BD elements may improve psychological balance and cognitive flexibility. Ultimately, circulation spaces should be redefined as active design components—architectural elements that mediate human restoration and adaptive thinking.

## 6. Conclusions

### 6.1. Principal Findings and Implications

This study advances both academic knowledge and practical application by empirically examining the effects of BD on learners’ neurophysiological responses within everyday circulation space—corridors and stairwells—in educational facilities. While previous research has primarily concentrated on static learning environments such as classrooms and libraries, the present findings demonstrate that even short-duration, high-mobility spaces can elicit cognitive activation and emotional stabilization when infused with BD. This suggests that areas traditionally regarded as mere passageways may be reinterpreted as restorative environments that facilitate psychological recovery and cognitive engagement.

The contributions of this study can be summarized in three points. Through verifying BD effects through objective physiological indicators (fNIRS and GSR), it extends neurophysiological approaches in architectural environmental research. Second, by identifying differential effects between corridors and stairwells, it expands the applicability of SRT and ART to circulation spaces. Third, by demonstrating that BD can promote emotional stability and cognitive vitality even in corridors, it provides actionable evidence to inform design strategies for healthier educational facilities.

### 6.2. Limitations

Nonetheless, this study acknowledges several limitations. First, it employed a relatively small and culturally homogeneous sample (N = 30), which constrains external generalizability [82]. All student participants and expert panel members were Korean. Because perceptions of BD—including aesthetic preference and restorative potential—can be culturally specific, this demographic homogeneity constitutes a notable limitation. Second, the reliance on image-based stimuli presents another limitation. While the use of Adobe Firefly was innovative for ensuring visual consistency and experimental control, image-based stimuli cannot fully replicate the embodied and multisensory dynamics of real built environments—particularly in spaces like stairwells, where movement, balance, and spatial depth are integral to experience. Although the AI-generated imagery achieved a high level of visual realism suitable for controlled testing, it may differ from the subtle material authenticity and multisensory cues of actual architecture. Moreover, potential stylistic bias from the generative model’s training data could slightly influence participants’ perceptual responses. Third, the analysis was restricted to two physiological measures, fNIRS and GSR, without incorporating a broader range of physiological or behavioral indicators, which limited the ability to fully capture the multidimensional mechanisms of BD effects. Finally, BD stimuli were presented only for a short duration, allowing for the assessment of immediate responses but not for sustained or cumulative effects under prolonged exposure.

### 6.3. Future Research

Future research should bridge the gap between static 2D imagery and immersive spatial experience by employing Virtual Reality (VR) and high-fidelity physical mock-up environments. In addition, studies should extend beyond short-term laboratory settings to examine long-term physiological responses, thereby enhancing ecological validity. Furthermore, considering individual differences—such as gender, academic level, baseline stress, and cultural background—may facilitate the development of more tailored BD strategies. Finally, by integrating multiple physiological and behavioral indicators, including heart rate variability (HRV), electroencephalography (EEG), and eye-tracking, future research could yield a more comprehensive understanding of the complex cognitive and emotional processes through which BD exerts its influence [83].

## Figures and Tables

**Table 1:** Experiences and Attributes of Biophilic Design by Stephen Kellert [20].

Experiences	Attributes
Direct Experience of Nature	Light/Air/Water/Plants/Animals/Landscape/Weather/ Views/Fire
Indirect Experience of Nature	Images/Materials/Texture/Color/Shapes and Form/Information Richness/Change, Age, and the Patina of Time/Natural Geometries/Simulated Natural Light and Air/Biomimicry
Experience of Space and Place	Prospect and Refuge/Organized Complexity/Mobility/Transitional Spaces/Place/Integrating Parts to Create Wholes

**Table 2:** The Effects of Biophilic Design in Educational Environments.

Ref.	BD Elements	Effects of BD
Terblanche and Khumalo [31]	Natural light, plants, views, natural materials	▪ Increased productivity ▪ enhanced mental, physiological, and psychological well-being
Alves, Betrabet Gulwadi and Nilsson [33]	Visual and non-visual interactions with nature, exposure to green spaces	▪ Support for connection with nature, others, and self ▪ mental recovery ▪ reduced fatigue
Mousighichi, Mousavi Samimi and Mousapour [36]	Physical, visual, and creative connections with nature; natural materials	▪ Increased place attachment ▪ improved physical, social health, and academic performance
Peters and D’Penna [34]	Natural light, views, plants, water features, natural materials, ventilation, etc.	▪ Reduced stress ▪ improved cognitive function and attention ▪ enhanced mood
Browning and Determan [29]	Natural views, biophilic patterns, natural light	▪ Reduced student stress ▪ improved academic performance
Determan, Akers, Albright, Browning, Martin-Dunlop, Archibald and Caruolo [37]	Natural light, views, plants, natural materials, biophilic and fractal patterns	▪ Improved academic performance ▪ reduced absenteeism and problematic behaviors ▪ increased teacher retention

**Table 3:** Biophilic Design Applications in Corridors and Stairwells of Educational Facilities.

Type	Images		Analysis	BDE *
	Corridor	Stairwell		
A	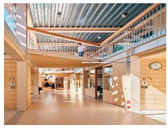	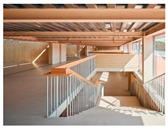	Natural materials and skylights provide abundant daylight. Creates a warm and dynamic circulation experience.	Weather & View, Material & Texture
B	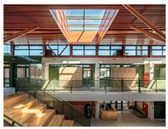	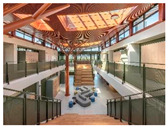	Wooden structures and transparent façades maximize daylight penetration. Central stairwell incorporates biomorphic forms.	Weather & View, Material & Texture, Forms & Shapes
C	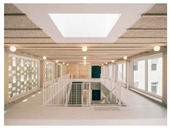	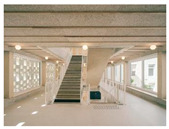	Open corridor and stairwell layouts enhance daylight utilization. Maintain continuous visual connection with the external environment.	Weather & View, Forms & Shapes
D	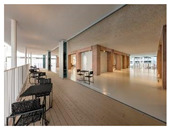	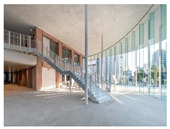	Curved glass façade ensures extensive daylight and outward views. Wood finishes enhance material warmth.	Weather & View, Material & Texture, Forms & Shapes

A. Kawasaki Korean School (Japan, 2024), photo by Kenya Chiba, architecture by KACH. B. Pinewood International School–Anatolia College (Greece, 2024), photo by Giorgos Sfakianakis, architecture by Micromega Architecture & Strategies & Alexandros N. Tombazis Architects. C. European University Center Refurbishment and Modernization, photo by Charly Broyez, architecture by Agence Vulcano-Gibello. D. KDU Campus Center, photo by Hiroyuki Oki, architecture by Atelier MEME. BDE *: Biophilic Design Elements.

**Table 4:** Seed images and default settings.

	Corridor	Stairwell
Seed image	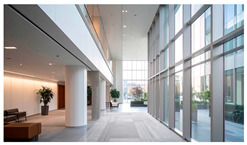	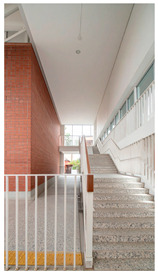
Prompt	modern university corridor, tall ceiling, large glass windows, minimalist design, white and grey tones	modern university stairwell, concrete stairs, white railings, red brick wall, large windows, bright natural light
Aspect ratio	16:9	9:16
Model	Firefly image 4	

**Table 5:** Parameter settings for image generation.

Parameters	Description	Value
Content type	A setting that determines the base style of the generated image.	Photo
Visual intensity	Adjusts how detailed, dramatic, and complex the generated image should appear.	6
Composition strength	Controls how closely the structure of the reference image should be followed.	2
Style strength	Controls how closely the style and level of detail of the reference image should be matched.	1

**Table 6:** Extracted keywords for prompt generation.

ELEMENTS	BD FRAMEWORK		Keywords
	Kellert [20]	Lee and Park [74]	
Weather & View	light, air, weather, views	light, views, landscapes	diffused/dappled natural light skylight atrium, clerestory window seasonal courtyard scenery, framed landscape views natural ventilation/breeze perception
Plants & Landscape	plants, animals, water, vitality	plants, animals, water	integrated green wall, vertical garden indoor water feature, reflective pool local vegetation/native species planting biodiversity-supporting habitats
Material & Texture	materials, texture	materials, color, simulated natural light	unfinished wood with visible grain, reclaimed stone, rattan, leather tactile-friendly handrails, textured wall panels warm, earthy color palette reflecting local ecology patina, natural wear for authenticity
Forms & Shapes	shapes and forms, natural geometries	shapes and forms, prospect, refuge	curved stairwell landing, rounded corners arched openings, biomorphic ceiling forms fractal skylight pattern, organic lattice structures prospect (long linear sightline), refuge (semi-enclosed alcove)

**Table 7:** Prompts for corridor and stairwell biophilic elements based on the biophilic design framework [20] and extracted keywords [74].

Areas	Elements	Subject	Attributes	Mood	Time & Background
corridor	Weather & View	floor-to-ceiling glazing, skylight atrium	diffused/dappled daylight, seasonal courtyard scenery, framed outdoor view	airy, harmonious, inviting	summer morning light, courtyard trees visible
	Plants & Landscape	integrated green wall, indoor aquarium wall (built-in, vertical)	vegetation, aquatic plants and fish visible in aquarium, shimmering reflections on glass	refreshing, restorative	summer greenery integrated indoors and outdoors
	Material & Texture	wood flooring, stone cladding, textured wall panels	unfinished wood grain, natural patina, tactile-friendly surfaces	warm, earthy, comfortable	neutral daylight enhancing natural textures
	Forms & Shapes	gently curved ceiling, arched corridor openings	organic geometries, biomorphic lines	natural, harmonious	daytime light highlighting ceiling patterns
Stairwell	Weather & View	skylight roof, clerestory windows	dynamic daylight transitions, cloud movement visible through glass	calm, contemplative	clear daytime sky, seasonal weather view
	Plants & Landscape	vertical green wall adjacent to stairwell, aquarium panel alongside steps	native plants, aquarium, biodiversity (fish, aquatic vegetation), dynamic light reflection from water surface	restorative, peaceful	indoor-outdoor connection with visible greenery
	Material & Texture	stone stair treads, wooden handrails	reclaimed stone surface, visible wood grain, tactile rail detail	grounded, stable, natural	daylight accentuating material authenticity
	Forms & Shapes	curved landing, semi-enclosed alcove seating, biomorphic skylight structure	fractal-inspired ceiling forms, organic lattice pattern, spatial depth (prospect) with refuge zones	safe, relaxing, immersive	midday natural light casting patterned shadows

**Table 8:** Biophilic design application simulation image.

Areas	Elements	Images	Prompts
Corridor	Weather & View	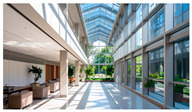	university corridor space, floor-to-ceiling glazing, skylight atrium, diffused/dappled daylight, seasonal courtyard scenery, framed outdoor view, airy, harmonious, inviting, summer morning light, courtyard trees visible
	Plants & Landscape	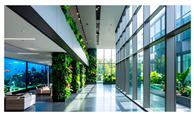	university corridor space, integrated green wall, indoor aquarium wall (built-in, vertical), vegetation, aquatic plants and fish visible in aquarium, shimmering reflections on glass, refreshing, restorative, summer greenery integrated indoors and outdoors
	Material & Texture	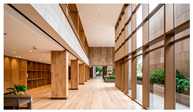	university corridor space, wood flooring, stone cladding, textured wall panels, unfinished wood grain, natural patina, tactile-friendly surfaces, warm, earthy, comfortable, neutral daylight enhancing natural textures
	Forms & Shapes	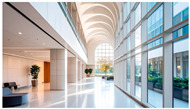	university corridor space, gently curved ceiling, arched corridor openings, organic geometries, biomorphic lines, natural, harmonious, daytime light highlighting ceiling patterns
Stairwell	Weather & View	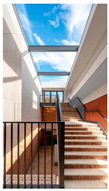	university stairwell space, skylight roof, clerestory windows, dynamic daylight transitions, cloud movement visible through glass, calm, contemplative, clear daytime sky, seasonal weather view
	Plants & Landscape	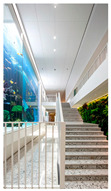	university stairwell space, vertical green wall adjacent to stairwell, aquarium panel alongside steps, native plants, aquarium biodiversity (fish, aquatic vegetation), dynamic light reflection from water surface, restorative, peaceful, indoor-outdoor connection with visible greenery
	Material & Texture	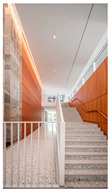	university stairwell space, stone stair treads, wooden handrails, reclaimed stone surface, visible wood grain, tactile rail detail, grounded, stable, natural, daylight accentuating material authenticity
	Forms & Shapes	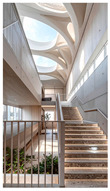	university stairwell space, curved landing, semi-enclosed alcove seating, biomorphic skylight structure, fractal-inspired ceiling forms, organic lattice pattern, spatial depth (prospect) with refuge zones, safe, relaxing, immersive, midday natural light casting patterned shadows

**Table 9:** Expert Evaluation Results for Visual Stimuli (Mean ± SD).

Areas	Elements	Evaluation Item				
		Architectural Authenticity	Contextual Appropriateness	BD Element Representation	Depth and Perspective	Visual Realism
Corridor	Weather & View	4.80 ± 0.45	4.80 ± 0.45	4.80 ± 0.45	4.80 ± 0.45	3.80 ± 0.45
	Plants & Landscape	4.60 ± 0.55	4.80 ± 0.45	4.60 ± 0.45	4.60 ± 0.55	3.60 ± 0.55
	Material & Texture	4.80 ± 0.45	4.60 ± 0.55	4.80 ± 0.55	4.80 ± 0.45	3.80 ± 0.45
	Forms & Shapes	4.80 ± 0.45	4.80 ± 0.45	4.80 ± 0.45	4.80 ± 0.45	4.00 ± 0.00
Stairwell	Weather & View	4.80 ± 0.45	4.80 ± 0.45	4.80 ± 0.45	4.80 ± 0.45	4.00 ± 0.00
	Plants & Landscape	4.60 ± 0.55	4.60 ± 0.55	4.60 ± 0.55	4.60 ± 0.55	3.60 ± 0.55
	Material & Texture	4.80 ± 0.45	4.80 ± 0.45	4.80 ± 0.45	4.80 ± 0.45	3.80 ± 0.45
	Forms & Shapes	4.80 ± 0.45	4.80 ± 0.45	4.80 ± 0.45	4.80 ± 0.45	3.80 ± 0.45

## Data Availability

The data presented in this study are available on request from the corresponding author. The data are not publicly available due to privacy.

## Abbreviations

The following abbreviations are used in this manuscript:

ART	Attention Restoration Theory
BA	Broca’s Area
BD	Biophilic Design
DLPFC	Dorsolateral Prefrontal Cortex
FPA	Frontal Polar Area
fNIRS	functional Near-Infrared Spectroscopy
GSR	Galvanic Skin Response
OFC	Orbitofrontal Cortex
SRT	Stress Reduction Theory
